# A *GNPTAB* nonsense variant is associated with feline mucolipidosis II (I-cell disease)

**DOI:** 10.1186/s12917-018-1728-1

**Published:** 2018-12-27

**Authors:** Ping Wang, Hamutal Mazrier, Jessica Caverly Rae, Karthik Raj, Urs Giger

**Affiliations:** Section of Medical Genetics, School of Veterinary Medicine, University of Pennsylvania, Philadelphia, PA USA

**Keywords:** Animal model, Cat, Lysosomal storage disease, N-acetylglucosamine-1-phosphotransferase, Mutation, Neuropathy

## Abstract

**Background:**

Mucolipidosis II (ML II; I-cell disease) is caused by a deficiency of N-acetylglucosamine-1-phosphotransferase (GNPTAB; EC 2.7.8.17), which leads to a failure to internalize acid hydrolases into lysosomes for proper catabolism of various substances. This is an autosomal recessive lysosomal storage disease and causes severe progressive neuropathy and oculoskeletal dysfunction in humans (OMIM 252500). A naturally occurring disease model has been reported in juvenile domestic cats (OMIA 001248–9685) with clinical signs similar to human patients. We investigated the molecular genetic basis of ML II in a colony of affected cats by sequencing the coding and regulatory regions of *GNPTAB* from affected and clinically healthy related and unrelated domestic cats and compared the sequences to the published feline genome sequence (NCBI-RefSeq accession no. XM_003989173.4, Gene ID: 101100231).

**Results:**

All affected cats were homozygous for a single base substitution (c.2644C > T) in exon 13 of *GNPTAB*. This variant results in a premature stop codon (p.Gln882*) which predicts severe truncation and complete dysfunction of the GNPTAB enzyme. About 140 *GNPTAB* variants have been described in human ML II patients*,* with 41.3% nonsense/missense mutations, nine occurring in the same gene region as in this feline model. Restriction fragment length polymorphism and allelic discrimination real-time polymerase chain reaction assays accurately differentiated between clear, asymptomatic carriers and homozygous affected cats.

**Conclusion:**

Molecular genetic characterization advances this large animal model of ML II for use to further define the pathophysiology of the disease and evaluate novel therapeutic approaches for this fatal lysosomal storage disease in humans.

**Electronic supplementary material:**

The online version of this article (10.1186/s12917-018-1728-1) contains supplementary material, which is available to authorized users.

## Background

Mucolipidosis II (ML II, OMIM 252500) is a hereditary lysosomal storage disease caused by the deficiency of N-acetylglucosamine-1-Phosphotransferase (GNPTAB, EC 2.7.8.17, previously known as GNPTA). This enzyme is a heterohexamer composed of three homodimers: alpha (α), beta (β) and gamma (γ) in a molar ratio of 2:2:2. It is responsible for placing an integral targeting signal, a mannose 6-phosphate group, on newly synthesized lysosomal acid hydrolases inside the Golgi apparatus. This modification facilitates the transfer of the acid hydrolases into the lysosome compartments for the degradation of substances, such as oligosaccharides, lipids, and glycosaminoglycans (GAGs) [1, 2]. Thus, GNPTAB deficiency is associated with a unique deficiency of multiple enzymes in lysosomes rather than one specific enzyme deficiency, which then leads to the storage of various waste products, such as mucolipids and GAGs, within lysosomes. Those cells with the storage granules, known as “inclusion cells” or “I-cells”, can be identified microscopically and lent the disorder its original name “I-cell disease” in humans [3]. There is an infantile to juvenile age of onset with rapid progression of clinical signs in human patients, leading to death within the first decade of life. The clinical features of ML II include stunted growth, skeletal joint abnormalities, coarse facial features, corneal clouding, mental retardation, hepatomegaly, cardiomegaly and respiratory infections, similar to mucopolysaccharidoses (MPS). A clinically milder form of ML, ML III (OMIM 252600, also called ML III α/β, ML IIIA or Pseudo-Hurler polydystrophy), also exists. It is characterized by later onset (childhood) and slower progression [4, 5]. Although mucolipidosis I and IV were historically classified as mucolipidosis diseases, they are currently no longer considered part of the mucolipidosis family. Nonetheless, these storage disorders, along with ML II and III, are often grouped as ML (Table 1).

**Table 1:** Classification of mucolipidosis in humans^a,b^ and animals^c^

Type	Synonyms	OMIM^a^	Gene	Enzyme/Protein deficiency	Major organ impairment	Animal species
I^d^	Sialidosis	256550	*NEU1*	α-N-acetyl neuraminidase	Skeletal & neurological	Mouse [8]
II	I-cell disease, ML II α/β	252500	*GNPTAB*	N-acetylglucosamine-1-phosphotransferase, α/β	Skeletal & neurological early onset, severe	Zebrafish^e^ [9, 10] Mouse^e^ [11–13] Cats, OMIA^c^ 001248–9685, [14–16]
III	Pseudo-Hurler polydystrophy, ML III α/β, ML IIIA	252600			Skeletal & neurologicallater onset, mild	None
	ML III γ, ML IIIC	252605	*GNPTG*	N-acetylglucosamine-1-phosphotransferase, γ		Zebrafish^e^ [17] Mouse^e^ [13, 18]
IV^d^	Sialolipidosis	252650	*MCOLN1*	Mucolipin-1 protein	Ophthalmologic	*Caenorhabditis elegans*^e^ [19]

^a^Online Mendelian Inheritance in Man (OMIM), https://www.omim.org

^b^Online Metabolic and Molecular Bases of Inherited Disease (OMMBID), https://ommbid.mhmedical.com

^c^Online Mendelian Inheritance in Animal (OMIA), https://www.omia.org

^d^Historically grouped with mucolipidoses

^e^Genetically engineered animal models

The three subunits (α, β and γ) of the GNPTAB enzyme complex are encoded by two genes. *GNPTAB* codes for the α and β subunits, and *GNPTG* codes for the γ subunit. In human patients, gene variants in *GNPTAB* cause ML II and ML III. Furthermore, *GNPTG* variants were discovered to also induce ML III clinically. Therefore, ML III was molecularly subtyped into an α/β form (ML IIIA) and a γ form (ML IIIC), where ML III α/β is due to *GNPTAB* pathogenic variants, while ML III γ is associated with variants in *GNPTG* [6, 7]. Currently, about 140 *GNPTAB* gene variants causing ML II and ML III α/β and 30 *GNPTG* variants causing ML III γ have been documented in human patients (The Human Gene Mutation Database, http://www.hgmd.cf.ac.uk).

In animals, ML II has been created in zebrafish [9, 10] and mice [11–13], and has been described as a naturally occurring disease in one domestic short-hair (DSH) cat from Switzerland (OMIA 001248–9685, ML II in *Felis catus*) [14, 15] which led to the clinicopathological characterization of the disease and the establishment of an animal colony at the School of Veterinary Medicine of the University of Pennsylvania [16]. No cases of ML III (either α/β or γ subtypes) have been documented to naturally occur in non-human species, but genetically engineered ML III γ disease models were created in zebrafish [17] and mice [13, 18].

Building upon initial work completed at the University of Pennsylvania which identified and characterized this naturally-occurring animal model of ML II, studies were undertaken to identify the molecular genetics of this disorder in the feline model. Those results are presented here.

## Results

### Clinical signs of ML II affected cats

One female (7160) and two males (7162 and 7163) out of five kittens born to clinically healthy DSH cats developed progressively deteriorating clinical signs shortly after birth. The three affected kittens showed very similar and typical clinical signs and disease courses, such as failure to thrive, coarse facial structures, short necks, ataxia, corneal clouding, behavioral dullness, and hepatomegaly. These severely progressive signs led to humane euthanasia within three months of life of affected kittens, while related carriers remained asymptomatic.

### Biochemical enzymatic diagnosis assessment

The activities of six lysosomal enzymes were markedly increased in serum from the three affected kittens (α-L-iduronidase, 7-fold; arylsulfatase B, 19-fold; β-glucuronidase, 24-fold; α-D-mannosidase, 10-fold; α-D-fucosidase, 16-fold and N-acetyl-β-D-glucosaminidase, 7-fold) compared to ten age-matched, healthy, unrelated control kittens (Table 2). The assay values from affected kittens for all six enzymes were statistically significantly increased (*p* < 0.01) from affected kittens when compared to unrelated control cats. Similarly, assay values for all six enzymes were also statistically significantly increased (*p* < 0.01) from affected kittens when compared to a combined group of unrelated control cats and clinically healthy littermates (n = total of 12). No statistically significant difference was found when comparing clinically healthy littermates to unrelated controls.

**Table 2:** Serum lysosomal enzyme activities in ML II affected kittens and clinical healthy littermates with normal ranges

Enzyme	Animal ID # and clinical status							Normal control^a^ Median (IQR), *n* = 10
	Affected				Healthy			
	7160	7162	7163	Median (IQR)	7161	7164	Median (IQR)	
	Activity^b^							
α-L-iduronidase	95.5	95.0	65.7	95.0 (29.8)^c^	14.8	13.7	14.3 (1.1)	14.0 (4.3)
arylsulfatase B	209.8	170.4	185.4	185.4 (39.4)^c^	10.9	9.9	10.4 (1.0)	9.8 (5.8)
β-glucuronidase	6126	7196	6061	6126 (1185)^c^	235.4	263.1	249.3 (27.7)	255.4 (29.3)
α-D-mannosidase	61,921	62,017	50,822	61,921 (11195)^c^	7079	6217	6648 (862)	6114 (3635)
α-D-fucosidase	2543	3015	2459	2543 (556)^c^	140.6	166.8	153.7 (26.2)	156.5 (44.2)
N-acetyl-β-D-glucosaminidase	9294	8380	6923	8380 (2371)^c^	1155	1015	1085 (140)	1138 (200)

^a^Reference values were based on 10 clinically healthy kittens, and presented as median with interquartile range (IQR)

^b^Enzyme activities were presented as nMol 4-MU/mL serum/hour

^c^*p* < 0.01 when compared to unrelated control cats and also to the combined group of unrelated control cats and clinically healthy littermates

Note, these enzyme activities were not measured in lysosomes (previously shown to be deficient [16]), and GNPTAB enzyme activity was not measured due to a lack of available substrate.

### *GNPTAB* gene sequence and nonsense variant identification

The feline *GNPTAB* gene is on chromosome B4 and consists of 21 exons with a coding sequence length of 1256 amino acid residues for the α and β subunits of the GNPTAB protein. The amino acid sequence homology of GNPTAB between felines (NCBI-ReqSeq accession no. XP_003989222.1, NCBI Genbank) and humans (NCBI-ReqSeq accession no. NP_077288, NCBI Genbank) is 91%.

**Fig. 1:**
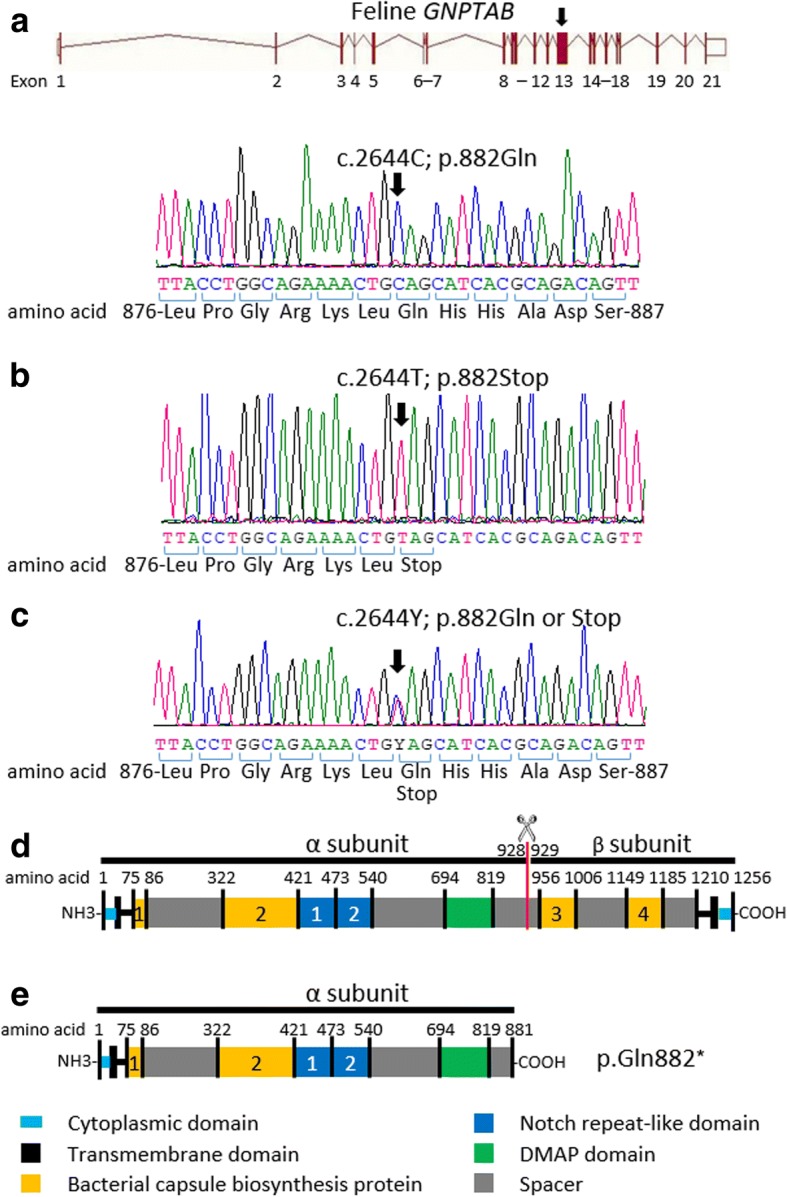
The feline *GNPTAB* structure and DNA sequencing results surrounding the c.2644C > T variation in exon 13 which is predicted to produce a GNPTAB protein without a β subunit. **a** A phenotypically healthy littermate (7161), showing homozygosity for cytosine (c.2644C, arrow). **b** A ML II affected kitten (7160), revealing homozygosity for a C > T substitution (c.2644 T), which will change the codon CAG (for glutamine) to TAG (a stop codon) at GNPTAB protein position 882 (p.Gln882*). **c** The clinically healthy tom cat (4977) of the ML II affected kittens, displaying both wild type and mutant allele (c.2644Y, heterozygous status). **d** Feline GNPTAB protein domain structure (adopted from human construct with 91% amino acids homology between human and cats). **e** The ML II affected cats with the p.Gln882* variant predicted to produce a GNPTAB enzyme with a truncated α subunit and without β subunit

Comparing the *GNPTAB* sequences of the three ML II affected kittens to an unrelated clinically healthy cat (9802) and published feline genome sequences, revealed one homozygous single nucleotide substitution in exon 13 c.2644C > T (Fig. 1). This base change results in the conversion of CAG, which is coding for glutamine (Gln), to TAG, a premature stop codon, at protein position 882 (p.Gln882*) causing truncation of the GNPTAB α/β-subunits. Furthermore, the clinically healthy parents of the affected kittens (tom 4977 and queen 6431) were heterozygous for the *GNPTAB* variant and wild type alleles while the clinically healthy littermates (7161 and 7164), which had normal serum lysosomal enzyme activities, and one clinically healthy unrelated cat (9802) were homozygous for the wild type allele. No other gene variants were found in the coding sequence of *GNPTAB* in affected kittens and their family members when compared to the published feline genome sequence of *GNPTAB*.

### Genotyping assays

**Fig. 2:**
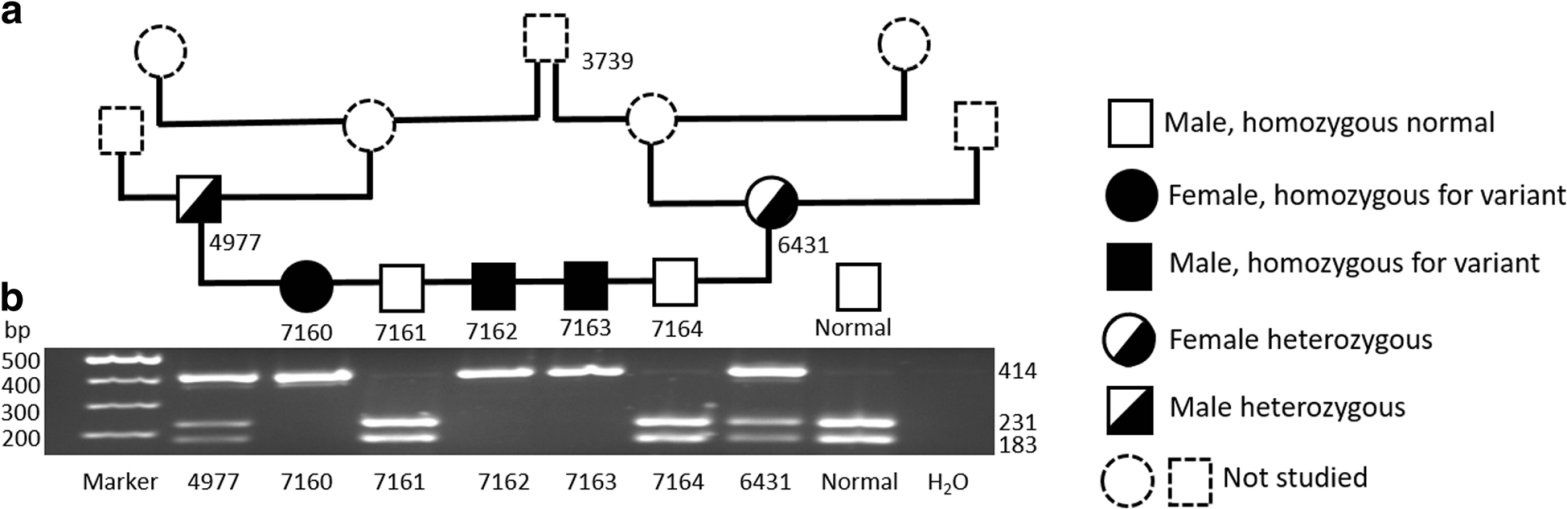
Pedigree and PCR-RFLP allele specific analyses within a family of the ML II affected cats. **a** Pedigree of a feline family descended from the first reported ML II affected cat [14, 15]. #3739 was the clinically asymptomatic half-brother of the originally affected cat. The family pedigree illustrates complete concordance and an autosomal recessive pattern. **b** Results of PCR-RFLP c.2644C > T allele specific analyses. A DNA fragment surrounding the variant was amplified by PCR, digested with PstI restriction enzyme, and visualized on 2% agarose gel stained with ethidium bromide. The amplified product is 414 bp in length, which includes one PstI digestion site. The clinically healthy kittens (7161 and 7164, and the normal control 9802) produced two fragments of 231 and 183 bp. The three ML II affected kittens (7160, 7162 & 7163) only showed an un-digested 414 bp fragment. The clinically healthy tom (4977) and queen (6431) were heterozygous for the wild type and variant alleles and therefore exhibited all three fragments of 414, 231 and 183 bp

In a restriction fragment length polymorphism (RFLP) genotyping assay, ML II affected kittens showed only the 414 base pairs (bp) uncut fragment, while their parents had three DNA fragments (carrier pattern; 414, 231 and 183 bp), and the two clinically healthy littermates and the clinically healthy unrelated cat (9802) revealed two fragments (183 and 231 bp) (Fig. 2b). Similarly, the mutant variant segregated completely in the family pedigree (Fig. 2a), confirming an autosomal recessive inheritance pattern.

The allelic discrimination real-time polymerase chain reaction (PCR) assay for the c.2644C > T nonsense variant in the *GNPTAB* gene clearly differentiated the three genotypes (Additional file 3 Figure S1): the ML II affected cats 7160, 7162 and 7163 were homozygous for the variant allele, presenting in the top left corner of the allelic discrimination plot; two clinically non-affected littermates (7161 and 7164) and one clinically healthy unrelated cat (9802) were homozygous for the wild type alleles, shown in the lower right corner of the plot; clinically non-symptomatic tom (4977) and queen (6431) exhibited the heterozygote genotype which appear in the center of the plot. Furthermore, there was complete segregation of the mutant *GNPTAB* allele among five ML II affected, eight obligate carriers and 50 healthy cats within the colony. Finally, 100 unrelated DSH cats from the local Pennsylvania region did not have the mutant *GNPTAB* allele, and no other naturally occurring cases of ML II have been reported since the original kitten.

## Discussion

This enzymatic and molecular genetic study of a related group of DSH cats clinically affected with ML II identifies the genetic defect causative of ML II to be a single nonsense variant in *GNPTAB*. Cats are the only naturally occurring large animal model of ML II identified to date, and have clinical features very similar to ML II in human patients, including failure to thrive, behavioral dullness, facial dysmorphia, retinal changes, decreased muscle tone, and ataxia, all of which are present at a young juvenile age (within two months of birth) with rapid progression. Thus, the molecular genetics, biochemical, and clinical abnormalities reported in these cats appear homologous with those of ML II in human patients [4, 5, 20], making this lineage of cats an ideal disease model to investigate therapeutic interventions.

Clinically, ML II presents similarly to other lysosomal storage diseases such as MPS, and the diagnosis must be confirmed by biochemical enzymatic and/or molecular genetic determinations [4]. Ideally, the biochemical diagnosis for ML II disease would directly detect the lack of GNPTAB activity in the white blood cells or cultured fibroblasts, but this enzymatic assay requires a radioactive substrate which is not commercially available. Instead, the diagnosis is typically reached by measuring the high activities of all lysosomal acid hydrolases in serum [16, 21]. This is because the pathogenic mechanism of ML II results in a defect in cellular trafficking of acid hydrolases, preventing intracellular transport of these enzymes into lysosomes, and resulting in the secretion of the enzymes extracellularly into the serum. In the study reported here, the enzymes from three cats with ML II were 7 to 24-fold above normal serum activities, values similar to what has been observed in human patients with ML II [21]. In previous work, we have demonstrated the lack of acid hydrolase enzymes in lysosomes of kittens with ML II [16].

Any gene variants affecting *GNPTAB* expression, secretion, and function could result in a lack of enzymatic GNPTAB activity and cause ML II or III α/β disorders. In this study of a family of cat with ML II, a single novel homozygous nonsense variant (c.2644C > T) in exon 13 was discovered in the *GNPTAB* coding sequence. This variant creates a premature stop codon (p.Gln882*).

The cleavage site of the α and β subunit precursor is located in exon 14 between residue 928, a lysine, and 929, an aspartic acid [22], in both humans and cats (Fig. 1d). It is predicted that the feline c.2644C > T, p.Gln882* nonsense variant would cause premature decay of the truncated messenger RNA of the GNPTAB α subunit, and a complete lack of the β subunit (Fig. 1e). The expected phenotype and pathogenic mechanism of action from a defective GNPTAB protein resulting from this truncated RNA message is consistent with the clinical signs and biochemical parameters observed in these cats. The clinical presentation and disease course due to this nonsense variant was completely penetrant in all affected ML II kittens in this study and very similar to previous reports [14–16].

While this p.Gln882* nonsense variant has not been described in human patients with ML II, nine nonsense/missense variants in this gene region (exon 13) have been documented in human patients with a severe ML II phenotype (Table 3), indicative of similar phenotype with mis- or nonsense variants in the same gene region. All nine variations have caused ML II with early progressive clinical symptoms in human patients [20, 23–27], similar to the feline disease model described here.

**Table 3:** Nonsense/missense variants identified in exon 13 of *GNPTAB* of humans and feline ML II patients

Species	HMD^a^ Accession #	cDNA	Protein	Reference
Human	CM100341	c.1759C > T	p.Arg587*	[20]
	CM144660	c.1774G > A	p.Ala592Thr	[23]
	CM101198	c.1875C > G	p.Phe624Leu	[24]
	CM094378	c. 1999G > T	p.Glu667*	[23]
	CM098787	c.2196G > T	p.Lys732Asn	[25]
	CM144659	c.2354 T > G	p.Lue785Trp	[23]
	CM053908	c.2533C > T	p.Gln845*	[26]
	CM100342	c.2664C > G	p.Tyr888*	[20]
	CM053913	c.2681G > A	p.Trp894*	[27]
Feline	NA	c.2644C > T	p.Gln882*	This study

^a^HMD: The Human Gene Mutation Database, http://www.hgmd.cf.ac.uk, accessed April 2018

Genotyping of ML II affected cats and their family members and comparison to unrelated cats confirmed that feline ML II is inherited as an autosomal recessive trait (Fig. 2a). Moreover, it verified the lack of the ML II genetic variant within the greater population of cats. Obligate and other carriers appeared clinically healthy but were readily detected by genotyping tests and were all related to the common ancestor. While this specific variation is not expected to be widespread in the feline population, the established RFLP and real-time PCR assays allow precise genotyping of animals in this research colony for assessment of novel therapeutic interventions.

This study builds upon a previously published abstract within which we reported preliminary molecular studies on *GNPTAB* in ML II cats [28]. At the time of the abstract’s publication, both the affected cat’s *GNPTAB* sequence and the feline genome reference sequence were incomplete. Complete sequencing of the affected cat’s *GNPTAB* exome, as reported here, as well as publication of the complete feline genome reference sequence, revealed that the *GNPTAB* missense variant referred to in the research abstract is, in fact, correctly a nonsense variant.

## Conclusion

Identification of the molecular basis of ML II in cats provides further evidence of its similarity to the human disease and enhances the use of this unique disease model to further define the pathogenesis of ML II and to evaluate safety and efficacy of novel therapeutic approaches for this lysosomal disorder in children.

## Methods

### Animals and phenotyping

Cats genetically-related to the originally-described cat with naturally-occurring mucolipidosis II identified in Switzerland [14, 15] have been a part of the animal colony at the School of Veterinary Medicine University of Pennsylvania for the past decade, and are cared for according to guidelines for animal research. The clinically non-affected half-brother (3739) of the originally affected cat was their common ancestor (Fig. 2a). A family (tom, queen, and five offspring including three ML II clinically affected kittens) was selected for this investigation. The clinically non-affected tom (4977) and queen (6431) produced five kittens with two clinically healthy (7161 and 7164 both male) and three ML II affected (7160 female, 7162 and 7163 both male) kittens. Unrelated age-matched (2–4 months) DSH cats kept in the same animal colony served as controls. Mucolipidosis II affected kittens were humanely euthanized at three months of age due to the severity of clinical manifestations. Euthanasia was performed with Euthasol euthanasia solution (Virbac AH, Inc., Fort Worth, TX, USA) at the dosage of 80 mg/kg intravenously according to the American Veterinary Medical Association Guidelines for the Euthanasia of Animals. The clinically asymptomatic cats remained in the colony or were adopted. The research methods and euthanasia protocol were approved by the Institutional Animal Care and Use Committee at the University of Pennsylvania (IACUC number A3078–01). Serum and ethylenediaminetetraacetic acid (EDTA) anticoagulated blood samples were collected from each cat for this study. Other DNA samples were obtained from a DNA bank at the University of Pennsylvania.

### Biochemical enzymatic diagnosis

The enzymatic activities of six lysosomal enzymes were measured in serum of affected and related kittens and compared to the enzymatic activities of ten age-matched clinically healthy unrelated cats. The tested enzymes assays were performed in duplicates for each sample according to the standard protocols utilizing the 4-Methylumbelliferone (4-MU) fluorometric endpoint method [29, 30] and as previously described by the PennGen laboratory [16] (Additional file 1). Enzyme activity values were expressed as nMol of 4-MU produced per mL of serum per hour.

Statistical analysis of the biochemical assay results from cats with ML II, unrelated control cats, and clinically healthy littermate, was performed. To overcome the non-heterogeneous range, the results were compared utilizing median values but not average values, using the Mann-Whitney U-test (two samples nonparametric tests, GenStat, version 18; VSN international ltd) [31]. *P*-values of less than 0.05 were considered significant. Results of the assays from cats with ML II were compared to those of the unrelated control cats for all six enzymes. Further analysis compared results from affected cats with a combined group of unrelated control cats and clinically healthy littermates, as well as comparison of unrelated control cats to clinically healthy littermates.

### Genomic DNA sequencing and variant identification

Genomic DNA (gDNA) was extracted from EDTA-anticoagulated blood from the tom and queen, and their five offspring, and a clinically healthy unrelated cat (9802) using QIAamp DNA Blood Mini kit (QIAGEN, Valencia, CA) following the manufacturer’s standard protocol.

The feline *GNPTAB* gene with its 21 exons as well as exon-intron boundaries, and its 5′- and 3′- untranslated regions, were amplified using 20 primer pairs and hot-start PCR (KOD Xtreme™ Hot Start DNA Polymerase kit, Novagen-EMD company, Gibbstown, NJ) following the laboratory’s established protocol [32]. The PCR primers were designed according to the published feline genome sequences (NCBI-RefSeq accession no. XM_003989173.4, Gene ID: 101100231, NCBI Genbank https://www.ncbi.nlm.nih.gov and Gene ID: ENSFCAG00000008281; Transcript ID: ENSFCAT00000008283.3, Felis_catus_6.2 reference genome assembly, Ensembl http://www.ensembl.org) and synthesized by Integrated DNA Technologies, Inc. (IDT, Coralville, IA) (Additional file 2). A negative control containing DNA-free water instead of gDNA was included in all assays to detect any possible contamination.

The PCR products were purified [32] and Sanger sequenced at the DNA Sequencing Core Facility of Perelman School of Medicine at the University of Pennsylvania: 6 μL (10 ng/μL) of template DNA for both forward and reverse primer tubes with 3 μL (1.1 μM) of each primer. The sequencing results were analyzed and compared to the published feline genome sequences as well as to the sequencing results of the clinically healthy cat (Lasergene software, DNAStar, Inc., Madison, WI).

### Genotyping assays

Based upon the discovery of a single nonsense variant in the *GNPTAB* gene in ML II affected cats, genotyping tests were established. For the RFLP assay, PCR primers surrounding the variant allele region (exon 13) were used (Additional file 2) which produced an amplicon of 414 bp followed by a PstI endonuclease (5′...CTGCA^G…3′, New England Biolabs, Ipswich, MA) digestion. Ten μL of the digested reaction mixture, which included PstI (10,000 U/mL), NEBuffer 3.1 (10X), and DNA-free water at a ratio of 0.5:2.0:7.5, was mixed with 10 μL of the PCR product in a 37 °C water bath overnight. The PCR product of the normal allele was digested into two fragments of 231 and 183 bp, which were visualized on a 2% agarose gel stained with ethidium bromide. The mutant variant allele removed the PstI recognition site and thereby prevented the digestion, resulting in a single 414 bp fragment.

For the allelic discrimination real-time PCR assay, reagents were obtained from the Custom Taqman SNP Genotyping Assay (Applied Biosystems, Life Technologies, Grand Island, NY). The DNA fragment around the nonsense variant was amplified by real-time PCR using a 15 μL reaction mixture containing 7.5 μL of 2X TaqMan Universal PCR master mix, 0.375 μL of 40X assay mix consisting of unlabeled PCR primers (Additional file 2), and fluorescently labeled TaqMan 3′-Minor groove binder probes (VIC dye-labeled CGTGATGCTGCAGTTT for wild type, and FAM dye-labeled CGTGATGCTACAGTTT for c.2644C > T substitution allele), 6.125 μL of DNA-free water, and 1 μL of template gDNA. Standard thermal cycling parameters were used: 95 °C for 10 min for an initial heat, 40 cycles of 15 s at 95 °C for denaturation and 60 s at 60 °C for annealing/extension, and 1 min at 60 °C for post-PCR hold. Results of amplification and detection were expressed as end-point fluorescence intensities (Rn values) plotted between wild type allele (X-axis) and variant allele (Y-axis) quadrants (Applied Biosystems ABI 7500 instrument, Life Technologies, Grand Island, NY).

The three ML II affected cats and their family members reported in this study (parents, obligate carriers [*n* = 2]; clinically asymptomatic littermates [n = 2]), and a clinically healthy unrelated cat were tested with both genotyping assays. An additional five ML II affected cats, eight obligate carriers, 50 healthy cats within the research colony and 100 unrelated DSH cats from the local Pennsylvania region were only tested by allelic discrimination real-time PCR assay.

## Additional files

Additional file 1: **Table S1.** Lysosomal enzymes assays: substrates, buffers, conditions and references. (DOCX 15 kb)

Additional file 2: **Table S2.** PCR primers and conditions used for DNA sequencing of the feline *GNPTAB* exons. (DOCX 25 kb)

Additional file 3: **Figure S1.** Allelic discrimination assay detects c.2644C > T variant in a cat family with ML II kittens. (TIF 2756 kb)

## Abbreviations

4-MU: 4-Methylumbelliferone
Bp: base pairs
DSH: domestic short-hair cat
EDTA: ethylenediaminetetraacetic acid
GAGs: glycosaminoglycans
gDNA: genomic DNA; Gln, glutamine
GNPTAB: N-acetylglucosamine-1-phosphotransferase
HMD: The Human Gene Mutation Database
IQR: interquartile range
ML II: mucolipidosis II
MPS: mucopolysaccharidoses
OMIA: Online Mendelian Inheritance in Animal
OMIM: Online Mendelian Inheritance in Man
OMMBID: Online Metabolic and Molecular Bases of Inherited Disease
PCR: polymerase chain reaction
RFLP: restriction fragment length polymorphism
