# Supplementary material for: A GNPTAB nonsense variant is associated with feline mucolipidosis II (I-cell disease)
Source: BMC Vet Res. 2018 Dec 27;14:416. doi: 10.1186/s12917-018-1728-1 (PMC6307278; doi:10.1186/s12917-018-1728-1)
Supplement: Supplementary file 1 — Table S1. Lysosomal enzymes assays: substrates, buffers, conditions and references. (DOCX 15 kb) [file 12917_2018_1728_MOESM1_ESM.docx]

**Additional file 1**

Table of lysosomal enzymes assays: substrates, buffers, conditions and references

| Enzyme | Substrate and concentration  (Vendor) | Reaction buffer | Serum (µL) | Reference |
| --- | --- | --- | --- | --- |
| α-L-iduronidase  (EC 3.2.1.76) | 4-MU* α-L-idopyranoside, 2 mM  (Toronto Research Chemicals, Canada) | 0.4 M Sodium formate  pH 4.4 | 15 | [28] |
| arylsulfatase B  (EC 3.1.6.12) | 4-MU sulfate potassium salt, 5 mM  (Glycosynth, Warrington, United Kingdom) | 50 mM Sodium acetate  pH 5.0 | 10 | [29] |
| β-glucuronidase  (EC 3.2.1.31) | 4-MU β-D-glucuronide, 10 mM  (Glycosynth, Warrington, United Kingdom) | 0.1 M Sodium acetate  pH 4.8 | 10 | [28] |
| α-D-mannosidase  (EC 3.2.1.24) | 4-MU α-D-mannopyranoside, 5 mM  (Sigma-Aldrich, St Louis, MO, USA) | 0.2 M Citrate phosphate  pH 4.4 | 1 | [28] |
| α-D-fucosidase (EC 3.2.1.51) | 4-MU α-L-fucoside, 1 mM  (Sigma-Aldrich, St Louis, MO, USA) | 0.2 M Citrate phosphate  pH 5.2 | 10 | [20] |
| N-acetyl-β-D-glucosaminidase  (EC 3.2.1.30) | 4-MU β-D-GlcNAc, 10 mM (Sigma-Aldrich, St Louis, MO, USA) | 0.2 M Citrate phosphate  pH 4.4 | 5 | [20] |

*4-MU: 4-Methylumbelliferone
