# Supplementary material for: A GNPTAB nonsense variant is associated with feline mucolipidosis II (I-cell disease)
Source: BMC Vet Res. 2018 Dec 27;14:416. doi: 10.1186/s12917-018-1728-1 (PMC6307278; doi:10.1186/s12917-018-1728-1)
Supplement: Supplementary file 2 — Table S2. PCR primers and conditions used for DNA sequencing of the feline GNPTAB exons. (DOCX 25 kb) [file 12917_2018_1728_MOESM2_ESM.docx]

**Additional file 2**

Table of primers and conditions of polymerase chain reactions used for DNA sequencing of the feline *GNPTAB* exons

| Exon | Sequence  (5’- 3’) | Tm  (^0^C) | Extension (sec) | Amplicon (bp) |
| --- | --- | --- | --- | --- |
| 1 | Forward: CAACCCAGCCTGCGGAGAG  Reverse: CCCAGAGCTGCAGGGAGGAT | 62 | 30 | 633 |
| 2 | Forward: TGGCATTGGGTGAATGGATGA  Reverse: CCCCCTTCAACCTGAACTGTTACAT | 60 | 30 | 324 |
| 3 | Forward: GGAAAAATGCGTGTAAGCTGAAGG  Reverse: TCAGTGCGGAACCTGATGTGG | 60 | 30 | 303 |
| 4 | Forward: TCATGGTTACTGTGGGAGGTATGTGA  Reverse: GACCAAGAGAACAGCGCACCA | 60 | 30 | 583 |
| 5 | Forward: CGAACGTTTGTGAGCATGTTGG  Reverse: GCAGCTCTTCTGCCTCTCTTGTT | 60 | 30 | 382 |
| 6 - 7 | Forward: AAATGCTTTGTAGTGTCCTGGCAGA  Reverse: ACACTGTGGCAGAATAGAGACCTGCT | 60 | 30 | 677 |
| 8 | Forward: TCAAACGTGTCTCTTCTTTGAGGTTGA  Reverse: AACCAATGGCAGTGATTGGAAAA | 58 | 30 | 354 |
| 9 - 10 | Forward: TCTGCCAGAGCCTGAGAGCA  Reverse: TCTCCTGAGCTTAGCACTCAAAACTGA | 60 | 45 | 806 |
| 11 | Forward: GGAATCCTACTTTGAGATTGGAACGTG  Reverse: GAGCCCGCTTTGGATGCTCT | 60 | 30 | 332 |
| 12 | Forward: TGCCCTTTGTTCAAGATGATAAGCA Reverse: CAAACTCCCCTCTACTCATCAAGGA | 58 | 30 | 358 |
| 13a | Forward: CAGCAGCTCTTGTGTTAGAATGTTTGA Reverse: TCCAGCGGGGCCTCTACACT | 60 | 45 | 838 |
| 13b | Forward: GGGTACAACCTGTCCAAGTCAGC  Reverse: TCCAATGGTTGGCACAGGAAA | 60 | 30 | 650 |
| 14 - 15 | Forward: GCACTCATTTCATTTGCCATTGTT  Reverse: TGGCACCAAAATGGACTAGTTGTG | 58 | 45 | 904 |
| 16 | Forward: CCACCCAGGTGCCCAGAAG  Reverse: TCCACTAGGCAGAAGAAAAGCAAGA | 60 | 30 | 380 |
| 17 - 18 | Forward: TTGCCTCACCCTGGTCTTGG  Reverse: CAAGCCACTTCCTTCTCTTGAGTCC | 60 | 30 | 651 |
| 19 | Forward: TCCCAACAGCTAGAAGGCACTTT  Reverse: CAGCAGATGTGCTTGGCCAGT | 60 | 30 | 554 |
| 20 | Forward: TCAAATTGCCTCCAGTGATTTTGTG Reverse: TCGACACCTGAGTTATATGCCAAAAGA | 58 | 30 | 353 |
| 21a | Forward: TGGGGAGGAATGATGGAGACTTG  Reverse: GGCCTCCACATGAAATCACATTC | 60 | 45 | 909 |
| 21b | Forward: TGTAAGTGGTCAACTACTGAGGAG Reverse: TCATCAGCTTCTGAGGTTCTTCCA | 58 | 45 | 1193 |
| 21c | Forward: GGGGGAAAATAAGTTCTAAATGGGAT Reverse: TTGTGCATTCTTGCTCTCTCAAAAA | 58 | 45 | 878 |
| Genotyping assays for disease causing variant in exon 13 | | | | |
| RFLP* | Forward: GAATCCACCCCCAGACTTAGAAAT  Reverse: Same as exon 13b Reverse above | 57 | 30 | 414 |
| Real-Time  PCR | Forward: CGATGTTAATGAAGTATTACCTGGCAGA  Reverse: GCAAAAAGCCCAGGTAACTGT | 60 | 60 | 68 |

*RFLP: Restriction fragment length polymorphism

Sources: (1) NCBI-RefSeq accession no. XM_003989173.4, Gene ID: 101100231, NCBI Genbank <https://www.ncbi.nlm.nih.gov> (2) Gene ID: ENSFCAG00000008281; Transcript ID:ENSFCAT00000008283.3, Felis_catus_6.2 reference genome assembly, Ensembl <http://www.ensembl.org>.
