# Supplementary figures and images for: A GNPTAB nonsense variant is associated with feline mucolipidosis II (I-cell disease)
Source: BMC Vet Res. 2018 Dec 27;14:416. doi: 10.1186/s12917-018-1728-1 (PMC6307278; doi:10.1186/s12917-018-1728-1)

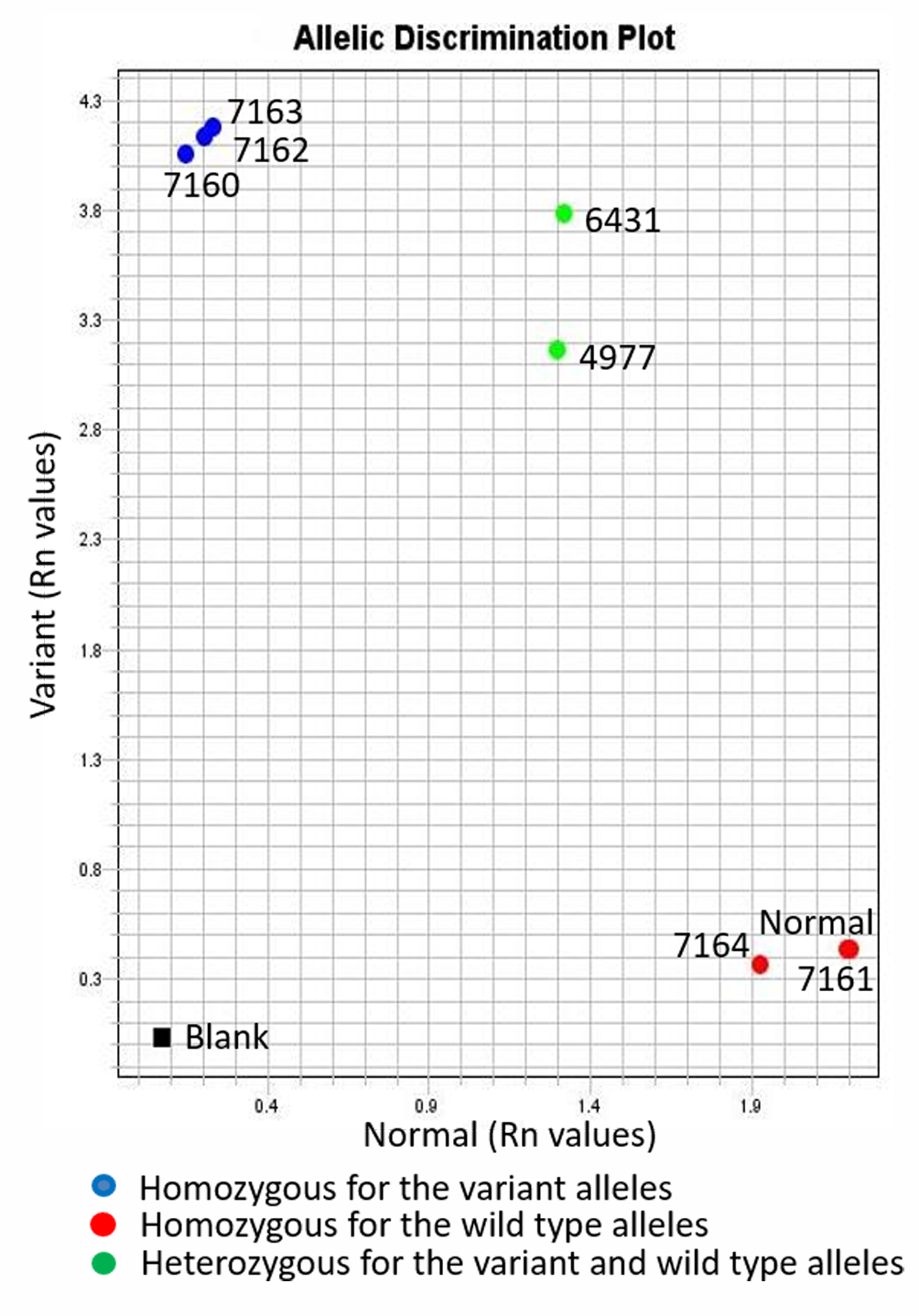

Supplement: Supplementary file 3 — Figure S1. Allelic discrimination assay detects c.2644C > T variant in a cat family with ML II kittens. (TIF 2756 kb) [file 12917_2018_1728_MOESM3_ESM.tif]
